# Investigating starch gelatinization through Stokes vector resolved second harmonic generation microscopy

**DOI:** 10.1038/srep45816

**Published:** 2017-04-06

**Authors:** Nirmal Mazumder, Lu Yun Xiang, Jianjun Qiu, Fu-Jen Kao

**Affiliations:** 1 Institute of Biophotonics, National Yang-Ming University, Taipei 11221, Taiwan; 2 Department of Biophysics, School of Life Sciences, Manipal University, Manipal, 567014, India; 3 Key Laboratory of Biomedical Photonics, Huazhong University of Science and Technology, Wuhan, China

## Abstract

The changes of the morphology during heating and the degree of crystallinity of dry and hydrated starch granules are investigated using second harmonic generation (SHG) based Stokes polarimetry. A spatial distribution of various polarization parameters, such as the degree of polarization (DOP), the degree of linear polarization (DOLP), and the degree of circular polarization (DOCP) are extracted and compared with the two dimensional second harmonic (SH) Stokes images of starch granules. The SH signal from hydrated and dry starch on heating differed significantly in DOLP and DOCP values, indicating that hydrated starch has a greater degree of ultrastructural amylopectin disorder. The detail of denaturation and the phase transition of hydrated starch demonstrate the significant influence of thermal processing.

In order to optimize the processing operations and obtain the desired quality of starch-based foods, a thorough understanding of the starch-water interaction through the gelatinization process is required^1^,^2^. The gelatinization is greatly influenced by the initial water content, the highest heating temperature, as well as the microstructures of starch granules^3^. Starch in the form of grains is the major storage compound in plants and so is an important part of our food^4^. Scientists are keen to understand the origins in plants, and how changes to the plants’ genes could affect the composition and properties of the starch in the grains^5^. The organization of shells has been widely studied using scanning electron microscopy on thin sections of granules^6^,^7^ nevertheless, detailed knowledge regarding the structure, organization and arrangement of lamellae is still limited. The finest features in the starch granule structure are due to the molecular packing of amorphous amylose and crystalline amylopectin lamellae^8^. Also, the sample preparation for the high resolution microscopy provides a complete structural elucidation of starch in its native form. The optical microscopy provides the detail of the microscopic structural information of starch non-invasively^9^.

Second harmonic generation (SHG) microscopy is an effective analytical tool for detailed investigation of microscopic structure of non-centrosymmetric molecules^10^. Stokes vector based SHG microscopy resolves the polarization states of the SH signal and allows the deduction of the molecular organization of collagen, skeletal muscle, starch etc.^11^,^12^,^13^. A strong SH signal from semi-crystalline amylopectin chains which are assumed to lie in the amorphous lamellae (amylose) form radially distributed amorphous growth rings in starch^14^,^15^. A Stokes vector based SHG microscopy scheme is distinct from several other previously demonstrated polarization resolved approaches in that it uses a large number of images rather than a single shot measurement^11^,^16^,^17^,^18^. The complete polarization states of the SH light of starch granules were characterized from SHG Stokes micrographs^11^,^13^. The technique was implemented to characterize the polarization properties of the SH signal from starch granule during starch-water interaction.

In pixel by pixel Stoke vector based SH image analysis, it is found that at room temperature the double helical amylopectin is self-organized upon hydration within starch granules^19^. In this article, the thermal behavior of these structurally complex materials is investigated by Stokes vector based polarization resolved SHG imaging. In addition, the chemical interactions between different components^20^ are observed from the reconstructed 2D SHG images using various polarization parameters, such as the degree of polarization (DOP), the degree of linear polarization (DOLP) and the degree of circular polarization (DOCP) from the acquired Stokes parameters^11^.

## Materials and Methods

### Sample preparation

The polarization-resolved SH images were obtained from potato starch in both dry (laboratory condition) and hydrated (suspended in water) condition using a Stokes-polarimeter. The starch granules were extracted from homegrown potatoes (additional details can be found in ref. 16). A drop of a dilute starch-water suspension was adhered to the coverslip and was placed on a round standard flexible thin heater (5 V, 10 mm inner diameter, temperature rise 100 °C, Taiwan KLC Corporation). A temperature programmer was connected to the heater and was controlled by changing the voltage from 20 to 100 °C. The heating plate was mounted upside-down in the microscope stage. The details of the temperature controller can be provided on request.

### Laser scanning microscope and data analysis

**Figure 1:**
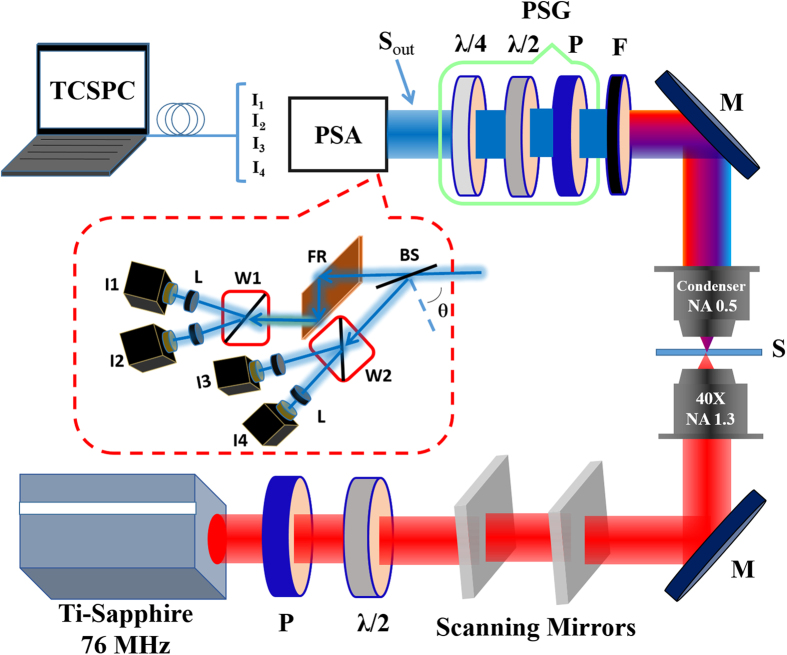
The schematic diagram of polarization resolved second harmonic generation four-channel Stokes-polarimeter setup. The setup was module based. PSG is inserted for the calibration of PSA and was removed afterward. λ/2: half wave-plate, λ/4: quarter wave-plate, S: sample, M: mirror, F: filter, BS: beam splitter, FR: Fresnel rhomb, W1 and W2: Wollaston prism, L: focusing lens, I_1_, I_2_, I_3_, I_4_: photo-multiplier tubes (PMTs), TCSPC: time correlated single photon counting.

The polarization properties of the SH signal are measured via four channel based SHG microscopy and the experimental arrangement is described in detail in ref. 11, 12, 13. A modified inverted Olympus IX81 confocal microscope is used as the imaging setup. The microscope consisted of a scan head (FV 300, Olympus) and a femtosecond Ti: Sapphire (Coherent Mira Optima 900-F) laser providing 100 fs pulses at 76 MHz tuned to a wavelength 800 nm. Samples were focused by an objective lens (UPlanFLN 40X/N.A. 1.3, Olympus Co., Japan) with a laser power of 3 mW and scanned with the laser scanning unit. The SH signal was detected at 400 nm in the forward direction using photomultiplier tubes (PMA 185, PicoQuant, Germany) and a combination of bandpass (400/40 nm, Chroma) and short pass (700SP, Chroma) filters. The polarization microscopy also includes a polarization state generator (PSG) and a polarization state analyzer (PSA). The various polarization states are generated using the PSG formed by a polarizer and a half wave plate. The SH signals were measured in transmission mode and analyzed by means of a polarization state analyzer (PSA), specifically, a schematics of four-channel Stokes-polarimeter is shown in Fig. 1.

The basic principles of four channel based Stokes polarimetry is discussed in detail in Mazumder *et al*.^11^,^12^. Fundamentally, polarization analysis can be carried out with Jones calculus, in which polarized light is expressed by the two-element Jones vector and the polarization elements are represented by the 2 × 2 Jones matrix. However, Jones calculus is limited only for perfectly polarized light and the complete polarization state cannot be revealed due to the lack of the phase difference between the two components of the measured polarization vector. For example, use of cross-polarized two-channel detection does not allow the relative ratio of the polarized and un-polarized components of the electric field to be determined^11^,^13^. For comparison, Stokes-Mueller formalism is a powerful approach that accommodates all polarization states, including incoherent, partially polarized, and unpolarized ones^11^,^20^,^21^.

The SH light can be completely characterized in terms of its intensity and state of polarization by the 4 × 1 Stokes vector 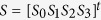, where the superscript *t* denotes the transpose of the matrix. The components of the Stokes vector are defined as below:


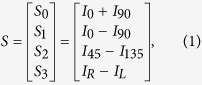


where *S*_0_ is the total intensity that corresponds to sum of the two orthogonal component intensities *I*_0_ and *I*_90_, *S*_1_ is the difference between the 0° (*I*_0_) and 90° (*I*_90_) polarization intensities, *S*_2_ is the difference between the +45° (*I*_45_) and −45° (*I*_135_) polarization intensities, and *S*_3_ is the difference between the left (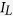) and right (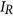) circular polarization intensities. These elements are generally normalized to the value of *S*_0_ so that their value ranges between +1 and −1.

The measured Stokes vectors of SH light can be expressed as S_out_ = A_4×4_^−1^.I where A_4×4_ is known as the instrument matrix of the polarimeter, S_out_ is the Stokes vector of the SH light, and I = [I_a_, I_b_, I_c_, I_d_]^t^, is the vector of the detected intensities measured in each arm of the polarimeter^11^,^21^. The four SH intensities (counts per msec) are detected simultaneously by time correlated single-photon counting electronics (TCSPC, PHR 800, PicoHarp300, PicoQuant GmbH, Berlin, Germany), as shown in Fig. 1. The 2D Stokes vector images ‘S_out_’ are reconstructed from the four SH signal intensity images acquired using a pixel dwell time of 8 μs with 256 × 256 pixels spatial resolution, which corresponds to a 50 × 50 μm scanning area. Data collection and primary analysis were achieved by a commercial software package (SymPhoTime 32, PicoQuant GmbH, Berlin, Germany). A series of MATLAB (MathWorks, R2009b) programs were developed to fully reconstruct the data.

Therefore, using Stokes matrix based formalism, crucial physical parameters of the SH light can be inferred from the measured Stokes parameters^11^,^12^,^13^, including DOP, DOLP and DOCP. These include the degree of polarization (DOP), degree of linear polarization (DOLP), degree of circular polarization (DOCP), as defined respectively by the following relations:


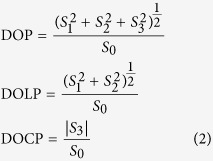


The physical significance of these parameters are indicates the polarization property of the light. If light is perfectly polarized, then DOP = 1 and 0 for an unpolarized light. For partially polarized light, DOP is in between 0 and 1, depending on the degree of polarization. The DOLP indicates the alignment of molecules parallel to the linear polarization states; DOLP is in between 0 and 1. The DOCP is a measure of how effectively the molecules flip the circularly scattered lights within the focal volume and the value ranges from 0 to 1.

## Results

### Stokes vectors dependency on Hydration and Dry of Starch Granules

**Figure 2:**
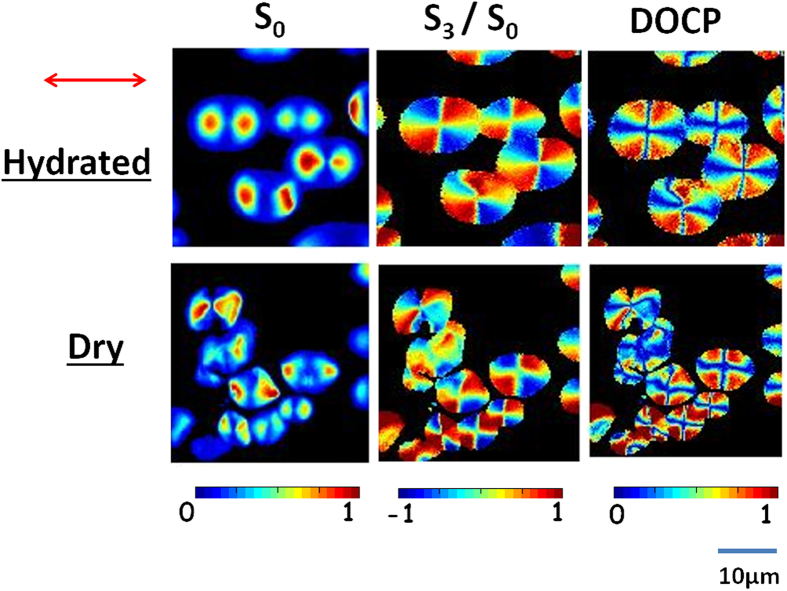
Compares the SH signal (S_0_), Stoke vector (S_3_) and DOCP of hydrated and dry starch granules. Experimental polarization-resolved 2D reconstructed Stokes vector and DOCP images of SHG response from hydrated and dry starch granule for the horizontal polarized excitation light at room temperature. The color scale shows the values of each parameter increases from blue to red.

We investigated the polarization states and reconstructed polarization parameters of SH light from both dry and hydrated starch in laboratory conditions. The SH signal depends on the geometrical characteristics and the relative phase difference of SH active molecules within the molecular arrangements^16^. From the observation of the electron diffraction data of potato starch fragments, Oostergetel and van Bruggen^1^ concluded that semi-crystalline domains form a network of left handed superhelices of diameter 18 nm and pitch 10 nm. It is consistent with previous reports identifying that amylopectin molecules in starch granules are the source of the SH signal^14^,^17^. It is noted that the strong SH signal arises due to the fraction of different handed amylopectin molecules being unequal, in turn producing a non-centrosymmetric structure^18^. The effect of the molecular arrangement of the concentric shell like structure of starch granules is observed in the SH intensity pattern and in various polarization components^12^,^17^. Figure 2 compares the SH signal (S_0_), Stoke vector (S_3_) and DOCP of dry and hydrated starch granules at room temperature. SHG images of dried potato starch granules were compared to the granules embedded in distilled water and a better behavior was observed for the hydrated polarization marker^22^. The dry starch granules are less intense than hydrated (S_0_) granules (data is shown in normalized form). The SH image of hydrated starch is more uniform than that of the dry starch, which is due to the increase in crystalline order of the starch granules during hydration^23^,^24^. Again, the difference in intensities (S_0_) are due to the scattering and crystalline order of the amylopectin during hydration. Recently, Richard *et al*. also found out the role of scattering by measuring the SH signal only of dry and hydrated potato starch^25^,^26^. Although there is an increase in SH intensity in hydrated starch but no significant change observed in the polarization parameters (S_3_ and DOCP). In another study, the image histogram of helical angle of SHG active molecule (amylopectin) remains unchanged under hydration^18^. Thus, the S_3_ and the DOCP which correlate to the helicity of the amylopectin molecules are unchanged during hydration compared to dry starch.

### Hydration and dry Starch Granules in heating conditions

**Figure 3:**
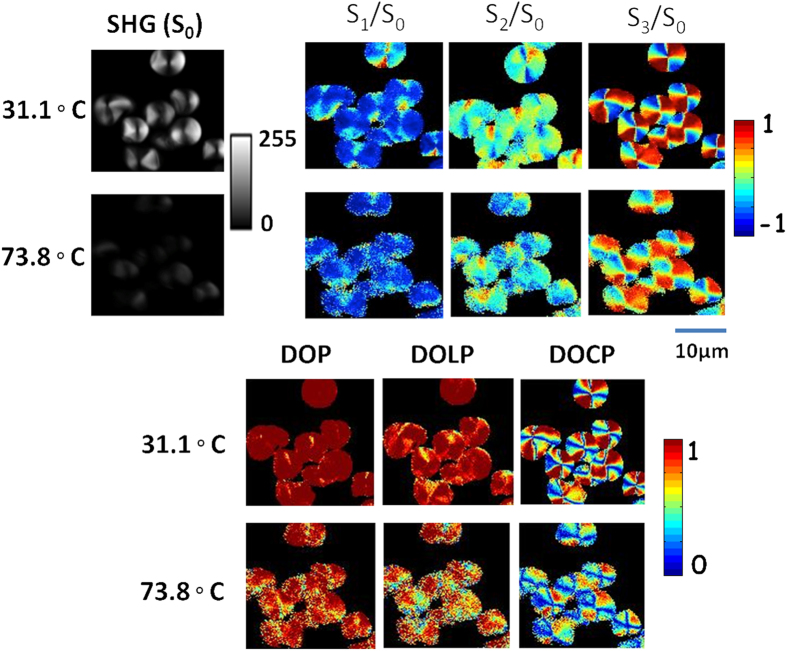
Micrographs of polarization-resolved SHG images. Experimental 2D reconstructed Stokes vectors (S_0_, S_1_, S_2_, S_3_), DOP, DOLP, and DOCP images of SHG response from hydrated starch granule for the horizontal polarized polarization respectively. The color scale shows the values of each parameter increases from blue to red. (Series of SHG images at various temperature is shown in Supplementary Fig. 1(a)).

**Figure 4:**
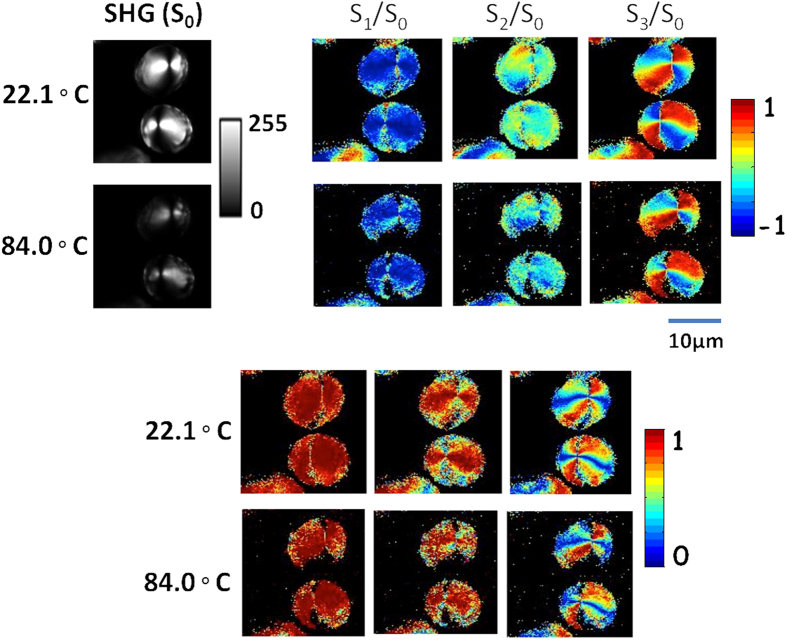
Micrographs of polarization-resolved SHG images. Experimental 2D reconstructed Stokes vectors (S_0_, S_1_, S_2_, S_3_), DOP, DOLP, and DOCP images of SHG response from dry starch granule for the horizontal polarized polarization respectively. The color scale shows the values of each parameter increases from blue to red. (Series of SHG images at various temperature is shown in Supplementary Fig. 1(b)).

The detail characterization of SH light from potato starch at different temperatures was performed using Stokes vector based polarimetry. Stokes vectors and polarization parameters are revealed in a pixel by pixel analysis which are visualized in the respective color bar as shown in Fig. 2. The heating conditions of hydrated starch were maintained in the microscope stage (as described in sample preparation). The images are acquired over ten min intervals for six different contrasts with a slow temperature ramp, sufficient photon counts were achieved to model the change in structure with temperature. Figures 3 and 4 compare the change of degree of crystallinity of dry and hydrated starch before and after heating.

The effect of temperature on the swelling power of starch acts differently^27^,^28^. The increase or decrease of swelling power at gelatinization temperature was caused by the transformation of amorphous amylose into a helical form, intermolecular interactions between amylose chains, interaction between crystallites and amorphous matrices^27^,^28^,^29^,^30^,^31^,^32^. When starch is heated to more than the critical temperature, an aqueous suspension of starch granules in excess water undergoes a co-operative endothermic transition known as gelatinization^31^ and thus the gradual disappearance of the crystallite responding to the loss of SH intensity at the phase transition temperature^25^. Figure 3 compares the SH signal (S_0_), Stokes vectors (S_1_, S_2_, S_3_) and DOP, DOLP, DOCP of hydrated starch granules at two different temperatures.

The SH signal and the respective Stokes vectors of dry starch granules are measured with increasing temperature. As shown in Fig. 4, there is no significant change of polarization parameters during heating. However, a small decrease in SH intensity (S_0_) of dry starch is observed at 84 °C, which is due to the presence of atmospheric water molecules inside the laboratory. Again, it is reported that in the case of dry starch granules, the hydroxide bonds are retained due to the loss of water and hydrogen bonds oriented closer to the helical axis of crystal domain^15^,^25^. There is negligible interaction between H-bonds in dry starch and no change of the polarization parameters (DOP, DOLP, and DOCP) are observed at high temperature.

## Discussion

The amylopectin lamellae are arranged in the A, B-type crystalline form in potato starch and^33^ hydration of these provides a significant effect on its structure. Researchers put forward a model for the self-assembly of amylopectin lamellae during hydration based on results from small and wide angle X-ray scattering^24^. Again, the effect of temperature on hydrated starch granules causes gelatinization and has been analyzed based on DSC (differential scanning calorimeter), small angle neutron scattering, dynamic mechanical analysis, optical microscopy and nuclear magnetic resonance data^34^,^35^. The molecular events responsible for this transition are not certain, but changes of shape and size of the granules, absorption of water and swelling, crystallite melting, and leaching of amylose (amylopectin) from the granules are observed^36^,^37^. Second-order nonlinear effects are, in particular, very sensitive to the structural symmetry of the samples; such that the anisotropic and concentric shell structure of starch granules gives rise to a unique polarization dependent behavior of the SH signal^7^,^12^. The break up and partial dissolution of amylopectin structure upon heating in the presence of water causes the degradation of the SH signal. We observed that at around 73.8 °C the SH signal diminishes (Fig. 3). At this temperature, the intermolecular bonds of hydrated starch molecules disrupt, allowing the hydrogen bonding sites to engage excess water. Therefore, progressively from B-type to C-type starch, crystallinity decreases rapidly with an increase in amylose content and destruct the average chain length of respective amylopectin^26^,^34^. At the same time, amylopectin believed to be covered by water molecules through H-bonds absorbed excess water and showed fundamental swelling during gelatinization, the result of solvation-assisted helix to coil transition while rapidly losing birefringence. The granules will not return to the initial structure when the starch dehydrated. The endothermic gelatinization processes are observed by DSC approximately at 65.4 °C (see Supplementary Fig. 2). The precise position of the peak depends on the diversity and composition of the starch structure being investigated^38^. Cooke and Gidley^32^ suggested that the enthalpy of gelatinization primarily reflected the loss of molecular (double-helical) order.

Figure 3 shows that the DOP values of the SH signal decreases at high temperature, i.e. the SH signal depolarizes. In Figs 3 and 4, both dry and hydrated starch, the DOLP and DOCP values are vice-versa. The higher the values of DOLP, the lower the DOCP value. Significant changes of degree of linear and circular birefringence (DOLP and DOCP) of the SH signal are observed in hydrated starch after heating, which is due to the destruction of anisotropic and helical structure inside the granule (Fig. 3). Again, in dry starch no significant change is observed up to 84 °C (Fig. 4). The changes of various polarization parameters of SH signal in dry and hydrated starch granules are significant and the evidence about self-assembly crystallinity of amylopectin lamellae are characterized using X-ray diffraction analysis (see Supplementary Fig. 3). Again, the structural morphology of starch granules are verified by scanning electron microscopy (SEM) image analysis (see Supplementary Fig. 4).

**Figure 5:**
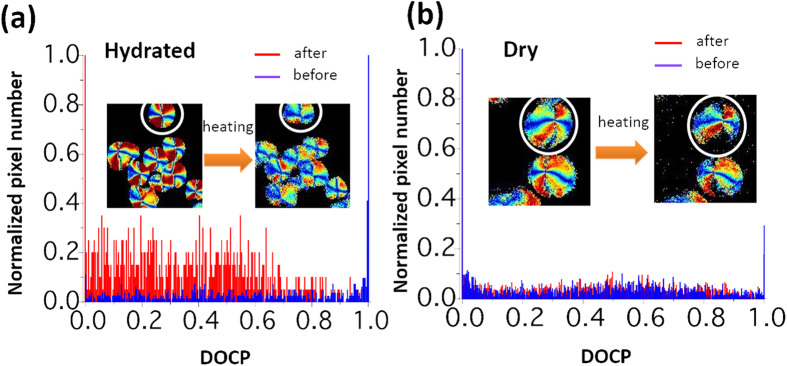
Histograms of polarization-resolved starch images. The DOCP histograms of (**a**) hydrated and (**b**) dry starch upon heating.

The origin of chirality in starch has been determined using second-harmonic-generation circular dichroism (SHG-CD) microscopy^17^ with high contrast and improved axial resolution. Figure 5 shows the pixel distribution of DOCP values in hydrated and dry starch upon heating. From the graph 5 (a), it is observed that the DOCP values are approximately 1 in all the pixels before heating (blue) and are lower than 1 after heating (red) of the hydrated starch granule. Again, for dry starch there is no significant change of DOCP pixel distribution during heating as shown in Fig. 5(b). This pixel analysis provides the spatial distributions of the helical structure of amylopectin inside the starch during the hydrated and dry condition.

## Conclusion

We investigated the starch gelatinization process through a Stokes vector based four-channel polarimeter integrated within a SH microscopy. The technique was shown to yield the polarization states of the SH signal, as quantified by the Stokes parameters, for a fixed input polarization state. Various polarization parameters of SH signals were calculated and analyzed, further elucidating structural properties of individual starch granules. The degradation of intermolecular integrally occurs in hydrated starch whereas disruption of surface in dry only. Thus, hydrated starch upon heating changes the polarization properties of the SH signal significantly, which suggests the deformation of crystallinity in starch granules. In the case of dry starch, there is no significant change of polarization properties observed at higher temperatures. Therefore, the fundamental understanding of the optical nonlinearity of starch granules will provide a valid basis for future studies in food science and insights into the energy transformation dynamics.

## Additional Information

**How to cite this article**: Mazumder, N. *et al*. Investigating starch gelatinization through Stokes vector resolved second harmonic generation microscopy. *Sci. Rep.* **7**, 45816; doi: 10.1038/srep45816 (2017).

**Publisher's note:** Springer Nature remains neutral with regard to jurisdictional claims in published maps and institutional affiliations.

## Supplementary Material

Supporting Information
