# Corrigendum: Investigating starch gelatinization through Stokes vector resolved second harmonic generation microscopy

**DOI:** 10.1038/srep46803

**Published:** 2017-05-16

**Authors:** Nirmal Mazumder, Lu Yun Xiang, Jianjun Qiu, Fu-Jen Kao

Scientific Reports 7: Article number: 45816; 10.1038/srep45816 published online: 04 06 2017; updated: 05 16 2017.

The original version of this Article contained an error in Affiliation 2, which was incorrectly given as:

‘Department of Biophysics, School of Life Sciences, Manipal University, Manipal, 567014, India’.

The correct affiliation is listed below:

‘Department of Biophysics, School of Life Sciences, Manipal University, Manipal 576104, India’.

This error has now been corrected in the HTML and PDF versions of the Article, as well as the accompanying Supplementary Information.

