# Supplementary material for: Investigating starch gelatinization through Stokes vector resolved second harmonic generation microscopy
Source: Sci Rep. 2017 Apr 6;7:45816. doi: 10.1038/srep45816 (PMC5382894; doi:10.1038/srep45816)
Supplement: Supporting Information [file srep45816-s1.pdf]

Supplementary information for Investigating starch gelatinization through Stokes vector resolved second harmonic generation microscopy

Nirmal Mazumder[1,2], Lu Yun Xiang[1], Jianjun Qiu[1,3], and Fu-Jen Kao[1]
[1]Institute of Biophotonics, National Yang-Ming University, Taipei 11221, Taiwan
[2]Present address - Department of Biophysics, School of Life Sciences, Manipal University, Manipal, 567014, India
[3]Key Laboratory of Biomedical Photonics, Huazhong University of Science and Technology, Wuhan, China

1. **Second harmonic generation (SHG) images of dry and hydrated starch granules at various temperatures:**

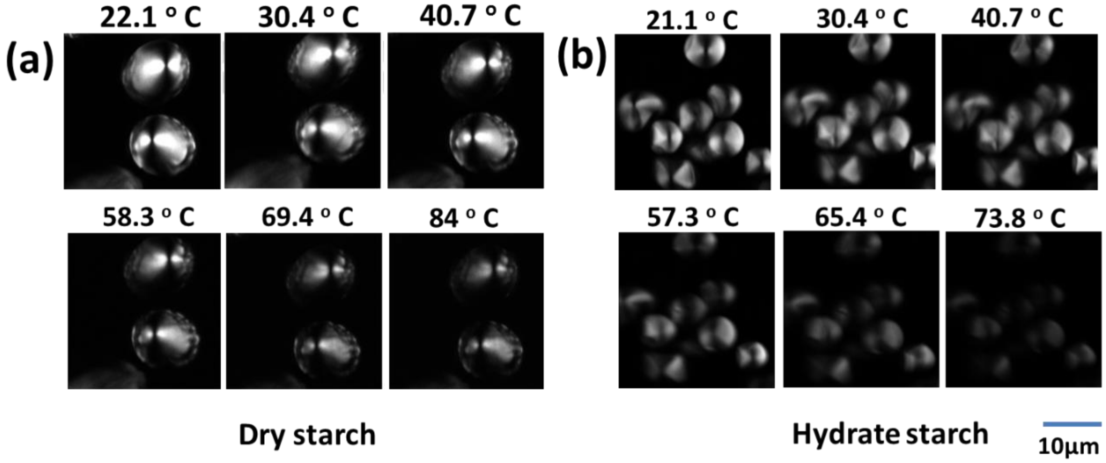

Supplementary Fig. 1: SHG images of a) dry and b) hydrated starch in heating conditions; a) the SH signal decreased at 84^{0}C and is comparable to 22.1^{0}C, b) SH signal decreased significantly at 73.8^{0}C, which is due to the destruction of degree of crystallinity.

2. **Differential Scanning Calorimetry (DSC) trace of native potato starch granules:**

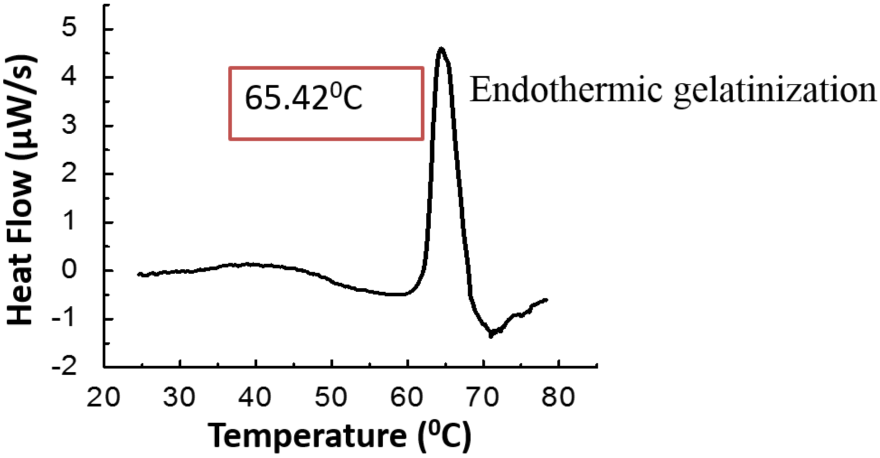

Supplementary Fig. 2: The Differential Scanning Calorimetry (DSC) trace of native potato starch shows an endothermic peak at the temperature 65.4^{0}C. This peak was also observed by other authors being assigned to the gelatinization of the material [1].

3. X-ray diffraction pattern of four types of starch granules:

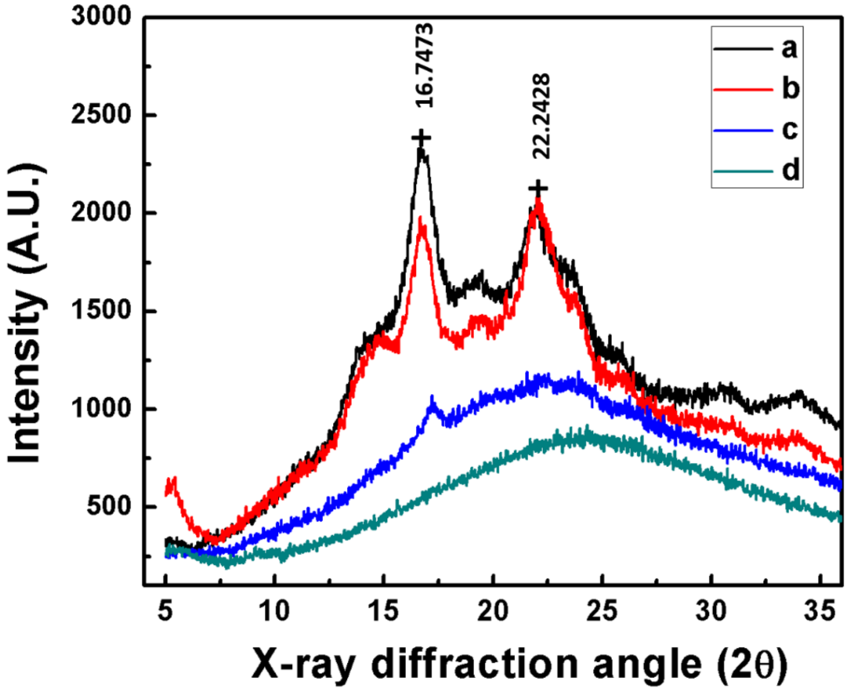

Supplementary Fig. 3: X-ray diffraction pattern of four types of starch (from Sigma Aldrich and homegrown potato); a) at room temperature (Sigma Aldrich, black); b) at room temperature (homegrown, red); c) at 84^{0}C (Sigma Aldrich, blue); d) at 74^{0}C (homegrown, light blue). Both the starch samples were heated for 15 min in a water bath with respective temperatures. The X-ray diffraction patterns of starch from Sigma Aldrich and natural starch granules at room temperature {black (a) and red (b)} have characteristic diffraction peaks near 17^0 and 23^0, indicating that two chemical structures are similar. The crystaline structure of both the starch granules are destroyed after heating and the amorphous nature is visibile in X-ray diffraction patterns {blue (c) and light blue (d)}. [2]

4. Scanning Electron Microscopy (SEM) of potato starch granules:

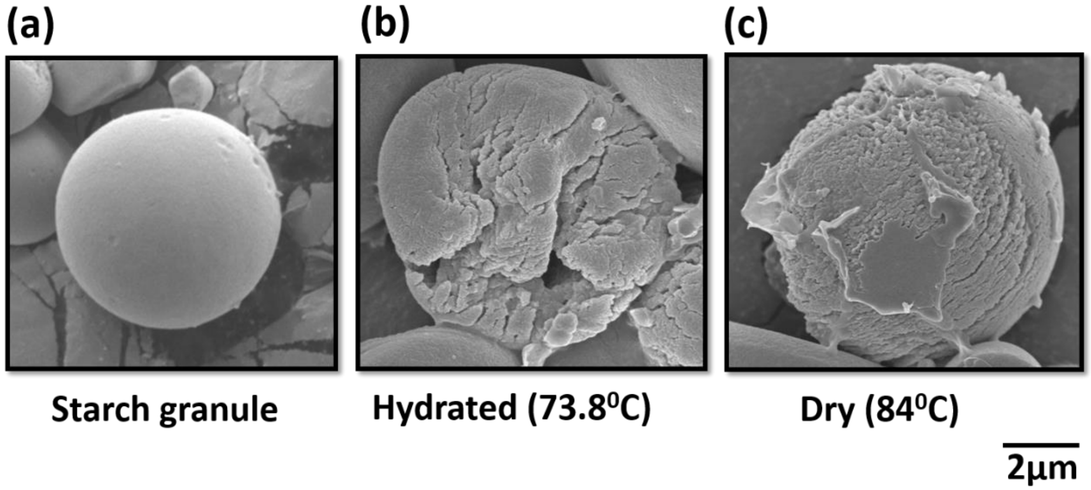

Supplementary Fig. 4: Scanning electron micrographs of potato starch granules; a) at room temperature, b) hydrated, c) dry. Micrographs a) shows the normal starch granule at room temperature without any degradation of structure, b, c) show the degradation of intermolecular integrally in hydrated starch whereas disruption of surface in dry.

References:

1. Donovan, J.W. Phase transition of the starch–water system *Biopolymers*, **1979,** *18*, 263–275.
2. Liu, Y.; Xu, Y.; Yan, Y.; Hu, D.; Yang, L.; Shen R. Application of Raman spectroscopy in structure analysis and crystallinity calculation of corn starch *Starch*, **2015**, *67*, 612–619.
